# Raising pH Reduces Manganese Toxicity in *Citrus grandis* (L.) Osbeck by Efficient Maintenance of Nutrient Homeostasis to Enhance Photosynthesis and Growth

**DOI:** 10.3390/plants14152390

**Published:** 2025-08-02

**Authors:** Rong-Yu Rao, Wei-Lin Huang, Hui Yang, Qian Shen, Wei-Tao Huang, Fei Lu, Xin Ye, Lin-Tong Yang, Zeng-Rong Huang, Li-Song Chen

**Affiliations:** College of Resources and Environment, Fujian Agriculture and Forestry University, Fuzhou 350002, China; raorongyu00@163.com (R.-Y.R.); huangwl1821993@163.com (W.-L.H.); 52308031045@fafu.edu.cn (H.Y.); 52308031061@fafu.edu.cn (Q.S.); 2220807009@fafu.edu.cn (W.-T.H.); 22308007010@fafu.edu.cn (F.L.); yexin1000@fafu.edu.cn (X.Y.); talstoy@fafu.edu.cn (L.-T.Y.)

**Keywords:** CO_2_ assimilation, manganese excess, nutrient homeostasis, OJIP transient, thylakoid structure

## Abstract

Manganese (Mn) excess and low pH often coexist in some citrus orchard soils. Little information is known about the underlying mechanism by which raising pH reduces Mn toxicity in citrus plants. ‘Sour pummelo’ (*Citrus grandis* (L.) Osbeck) seedlings were treated with 2 (Mn2) or 500 (Mn500) μM Mn at a pH of 3 (P3) or 5 (P5) for 25 weeks. Raising pH mitigated Mn500-induced increases in Mn, iron, copper, and zinc concentrations in roots, stems, and leaves, as well as nitrogen, phosphorus, potassium, calcium, magnesium, sulfur, copper, iron, and zinc distributions in roots, but it mitigated Mn500-induced decreases in nitrogen, phosphorus, potassium, calcium, magnesium, sulfur, and boron concentrations in roots, stems, and leaves, as well as nutrient imbalance. Raising pH mitigated Mn500-induced necrotic spots on old leaves, yellowing of young leaves, decreases in seedling growth, leaf chlorophyll concentration, and CO_2_ assimilation (A_CO2_), increase in root dry weight (DW)/shoot DW, and alterations of leaf chlorophyll *a* fluorescence (OJIP) transients and related indexes. Further analysis indicated that raising pH ameliorated Mn500-induced impairment of nutrient homeostasis, leaf thylakoid structure by iron deficiency and competition of Mn with magnesium, and photosynthetic electron transport chain (PETC), thereby reducing Mn500-induced declines in A_CO2_ and subsequent seedling growth. These results validated the hypothesis that raising pH reduced Mn toxicity in ‘Sour pummelo’ seedlings by (*a*) reducing Mn uptake, (*b*) efficient maintenance of nutrient homeostasis under Mn stress, (*c*) reducing Mn excess-induced impairment of thylakoid structure and PEPC and inhibition of chlorophyll biosynthesis, and (*d*) increasing A_CO2_ and subsequent seedling growth under Mn excess.

## 1. Introduction

Manganese (Mn) is not only an essential micronutrient for plants but also a heavy metal (HM) that is detrimental to plants when soil Mn is excessive [1]. Mn toxicity is primarily expressed in plant shoots, characterized by reduced growth, chlorosis, and necrosis in leaves [2]. The amount of Mn^2+^ (the most available form for plants) in the soil solution augments with the decrease in pH [3]. With the increase in Mn availability in the soil, the accumulation of Mn in plants increases dramatically, which easily exceeds the plant’s requirement for Mn. Therefore, Mn toxicity often occurs in plants, especially in acidic soil with a pH < 5.0 [4]. Mn toxicity has been suggested to be the second metal toxicity on acidic soils after aluminum (Al) toxicity [2].

After Mn is absorbed by roots, most of Mn is transported from the roots to the shoots and further transported to various tissues to meet growth needs [5]. Excessive Mn inhibits the biosynthesis of chlorophyll (Chl) and increases the accumulation of oxidized Mn in leaf apoplast, thus causing chlorosis in young leaves and necrosis (brown speckles) in old leaves [5,6]. Brown speckles on mature leaves are common signs of Mn toxicity. These brown speckles contain oxidized Mn, but the brown color derives from oxidized polyphenols rather than Mn [1]. Qiu et al. [7] investigated the effects of Mn excess on four citrus rootstocks, namely, ‘Ziyang Xiangcheng’ (*Citrus junos* Sieb. ex Tanaka), trifoliate orange (*Poncirus trifoliata* (L.) Raf.), ‘Shatian pummelo’ (*Citrus grandis* (L.) Osbeck), and red tangerine (*Citrus reticulata* Hort. ex Tanaka). The common Mn-toxic symptoms of citrus included stunted (reduced root and shoot) growth, increased root dry weight (DW)/shoot DW (R/S), chlorosis and brown necrotic spots in leaves, and brown spots in roots. Mn toxicity causes a significant impact on chloroplast ultrastructure [6,8,9]. Papadakis et al. [8] found that excess Mn impaired the chloroplast ultrastructure of *Citrus volkameriana* L. plants, characterized by reduced growth of grana, increased presence of starch grains, and increased depositions of dark materials in thylakoids. Excessive Mn disrupted the chloroplast ultrastructure of peach (*Prunus persica* (L.) Batsch) plants (swelling of the spaces between thylakoids, granum deformation, and increased number and size of plastoglobules) by increasing oxidative damage [6]. Chloroplasts from Mn-exposed pecan (*Carya illinoensis* K. Koch) plants were thinner and smaller than those of control plants, but chloroplast structure was kept intact [9].

Many studies have shown that excessive Mn inhibits CO_2_ assimilation (A_CO2_) in many plants, including pummelo [7,10], trifoliate orange [7,11], ‘Ziyang Xiangcheng’, red tangerine [7], peach [6], *Acer mono* Maxim., *Ulmus davidiana* Planch., *Betula ermanii* Charm., and *Alnus hirsuta* Turcz. ex Rupr. [12]. Noor et al. [6] observed that Mn excess caused a reduction in both A_CO2_ and C_i_ in peach leaves, implying that the reduction in A_CO2_ was at least partially caused by stomatal closure. However, other studies suggested that the reduction in A_CO2_ in pummelo, trifoliate orange, ‘Ziyang Xiangcheng’, and red tangerine leaves caused by Mn excess was primarily due to non-stomatal factors, because intercellular CO_2_ concentration (C_i_) was increased by Mn excess [7,10,11]. The reduction in A_CO2_ in ‘Sour pummelo’ leaves in response to Mn excess was not caused by the reduction in Chl concentration, because the Chl concentration was not significantly affected by Mn excess [10]. However, Mn excess-induced reduction in Chl concentration in peach leaves was not lower than that of A_CO2_ [6]. The reduction in photosynthesis in wheat leaves caused by excessive Mn was due to the peroxidative damage of the thylakoid membrane function [13]. St Clair and Lynch [14] reported that Mn excess reduced the photosynthetic capacity of red maple (*Acer rubrum* L.) and sugar maple (*Acer saccharum* Marsh.) leaves, particularly under high light, but quantum yield of photosystem II (PSII)/quantum yield of CO_2_ fixation and antioxidant enzyme data suggested that the reduction in the photosynthetic capacity was not due to photo-oxidative damage. Li et al. [10] associated the Mn excess-induced reduction in A_CO2_ in ‘Sour pummelo’ leaves with the impaired photosynthetic electron transport chain (PETC). However, net photosynthetic rate in white birch (*Betula platyphylla* Sukaczev) leaves reduced with leaf Mn accumulation, but maximum quantum yield of primary photochemistry (F_v_/F_m_) was little affected by Mn accumulation [15]. To conclude, the mechanism by which excessive Mn causes a reduction in leaf A_CO2_ is still poorly understood.

Excessive Mn can disrupt root architecture and function, reduce root growth, and interfere with the uptake and translocation of other nutrients [1,6,7,14,16,17,18,19,20]. Excessive Mn is often accompanied by an induced lack of magnesium (Mg) [1,7], calcium (Ca) [1,7,20], iron (Fe) [1], phosphorus (P) [6,20], and potassium (K) [20]. In berseem (*Trifolium alexandrinum* L.), excessive Mn reduced the concentrations of K, Ca, and P in roots, stems, and leaves [20]. In soybean (*Glycine max* L.), excessive Mn reduced Fe concentration in leaves but increased Fe concentration in roots, and excessive Mn reduced the concentration of Mg more in leaves than in roots [16]. In wheat (*Triticum aestivum* L.), excessive Mn decreased the concentrations of Mg and Ca in shoots but did not affect their concentrations in roots [18]. In ‘Ziyang Xiangcheng’, trifoliate orange, ‘Shatian pummelo’, and red tangerine, the concentrations of K, P, Fe, and zinc (Zn) generally declined in leaves and increased in roots with the increase in Mn supply; the concentrations of Mg and Ca in roots and leaves declined with the increase in Mn supply; and Mn excess led to a greater reduction in root Ca concentration than in root Mg concentration [7]. In sugar maple (*Acer saccharum* Marsh.), Mn excess reduced the concentrations of K and Fe in young and mature leaves and of Ca in young leaves but did not affect the Zn and Mg concentrations in young and mature leaves and of Ca in mature leaves. In red maple (*Acer rubrum* L.), Mn excess did not alter the concentrations of Ca, Mg, K, Fe, and Zn in young and mature leaves with the exceptions that Mn reduced the concentrations of Mg, Ca, and Fe in mature leaves and of Zn in young leaves [14]. In perennial ryegrass (*Lolium perenne* L.), excessive Mn led to a strong reduction in the concentrations of Mg and Ca in the shoots of the Mn-sensitive genotype, but only slight variations in the Mn-resistant genotype [19]. Thus, it seems that the influence of Mn excess on the uptake and translocation of other nutrients depends on plant species, genotypes (varieties), and Mn concentration. Notably, all these studies did not examine the effects of Mn excess on nitrogen (N), boron (B), copper (Cu), and sulfur (S) uptake. Mn excess-induced lack of Mg and Fe is associated with inhibited absorption across the plasma membrane and competition (or imbalance) at the cellular level, and Mn excess-induced deficiency of Ca is suggested to be an indirect impact on the Ca transport to expanding leaves [1]. Mn excess-induced inhibition of Mg and Fe uptake can damage chloroplast structure and reduce Chl biosynthesis and photosynthesis [17]. In peach seedlings, P reduced Mn uptake under Mn excess, prevented Mn toxicity-induced impairment of chloroplast ultrastructure, and improved leaf A_CO2_ and plant growth under Mn excess [6,21]. Mn excess can often be alleviated through application of Mg [1,22]. Exogenous application of Mg reduced Mn toxicity in tomato (*Lycoversicon esculentum* Mill.) by inhibiting Mn uptake, increasing the Mg/Mn ratio in shoots (leaves), and counteracting the onset of Mn toxicity in shoots (leaves) with high Mn concentrations [23]. Application of Mg conferred wheat Mn tolerance by repressing Mn uptake, increasing the Mg/Mn ratio, and reducing root-to-shoot Mn transport [24]. Mg/Mn in shoots (leaves) is suggested to be a better indicator of Mn toxicity symptoms than Mn concentration alone [23]. Liu et al. [25] showed that the ameliorative effect of H_2_S on Mn excess in *Malus hupehensis* (Pamp.) Rehder plants was caused by a combination of factors, including (*a*) reduced Mn uptake and increased Ca, Mg, Fe, and Zn uptake under Mn excess and (*b*) elevated Chl concentration, A_CO2_, and growth under Mn excess. Fe ameliorated Mn toxicity in sunflower (*Helianthus annuus* L.) and soybean plants by lowering Mn concentration in leaves [26].

To deal with Mn toxicity, plants have developed various adaptive strategies. Increased fixation of Mn in Mn-exposed roots is suggested to be an adaptive mechanism of plants to Mn excess [27]. Mn-tolerant *Citrus sinensis* (L.) Osbeck seedlings displayed a higher Mn distribution in roots and lower Mn concentration in leaves than Mn-sensitive *Citrus grandis* (L.) Osbeck seedlings under excessive Mn [28,29]. Mn-tolerant ryegrass genotypes accumulated more Mn in roots than sensitive ones [19]. Decreased uptake of Mn is another adaptive mechanism of plants to Mn excess [5]. Zheng et al. [3] indicated that decreased Mn absorption via rhizosphere alkalization and secretion of low-molecular-weight compounds by roots was responsible for the high Mn tolerance of *Citrus sinensis* (L.) Osbeck seedlings. The alleviation of Mn toxicity in trifoliate orange mediated by arbuscular mycorrhizal fungi involved reduced Mn uptake [11]. Overexpression of the *Stylosanthes guianensis* (Aubl.) Sw. malate dehydrogenase1 gene (*SgMDH1*) conferred transgenic *Arabidopsis* plants Mn tolerance through increasing root malate secretion and lowering plant Mn level [30]. Silencing of the *Prunus persica* (L.) Batsch Natural Resistance Associated Macrophage Protein 5 gene (*NRAMP5*) conferred Mn tolerance in peach by reducing the Mn concentration in leaves and roots [31].

Traditionally, Mn toxicity can be corrected by increasing pH [32]. It is imperative to reveal the mechanism by which increasing pH reduces Mn toxicity in plants to understand their adaptation to acidic soils with high Mn availability. To our knowledge, such studies are very limited, since low pH and Mn toxicity were investigated separately [4,33,34]. In a study, Rosas et al. [35] reported that pH 6.0 alleviated Mn toxicity-induced decrement in white clover (*Trifolium repens* L.) shoot and root dry weight (DW) and increased root Mn concentration relative to pH 4.8 but had little impact on leaf Mn concentration and that the concentrations of P and Fe were higher at pH 6.0 than at pH 4.8, which might be the cause for the differences in shoot and root DW obtained at different pH values. Unfortunately, this study did not investigate the effects of Mn-pH treatments on other nutrients (N, Ca, S, Mg, K, B, Cu, and Zn) and photosynthetic performance. By applying NaHCO_3_ to increase the pH, the Mn toxicity of peanut (*Arachis hypogaea* L.) was reduced, while the leaf Mn level was lowered and the leaf Mg/Mn ratio was increased [22]. All these studies, however, have focused on phenomena occurring on herbaceous plants. To our knowledge, there is no report on the increased pH-mediated alleviation of Mn toxicity on woody plants. Recent studies indicated that in ‘Xuegan’ (*Citrus sinensis* (L.) Osbeck) seedlings, increasing pH mitigated Al (Cu) toxicity-induced increment in Al (Cu) uptake and impairment to nutrient homeostasis and leaf PETC and increased leaf A_CO2_ and subsequent seedling growth under Al (Cu) toxicity [36,37]. This drives us to believe that increasing pH can also alleviate the toxicity of Mn in woody (citrus) plants in this way.

Citrus trees are mainly cultivated on red earth, yellow soil, or latosol in southern China, where acidification is becoming increasingly severe and the concentration of available Mn is high. In some citrus orchards with acidic soils, Mn toxicity and low pH often coexist [38]. Wu et al. [39] analyzed the pH and available Mn of 2374 soil samples and the Mn concentration of 2087 leaf samples from main citrus-producing areas in China. They found that 49.1% and 47.5% of soil samples had a pH < 4.8 and an available Mn concentration above the sufficient range, respectively, and 51.2% of leaf samples had an Mn concentration above the sufficient range. Therefore, it is necessary to examine the impacts of Mn-pH treatments on citrus plants so as to reveal the adaptive mechanism of citrus plants to acidic soils with high Mn availability and to provide a theoretical basis for developing a novel method to combat citrus Mn toxicity. Previously, some researchers investigated the effects of Mn excess on growth [7,10], uptake of Mn [3,7,10,11] and other nutrients [7], rhizosphere alkalization and secretion of low-molecular-weight compounds by roots [3], carbohydrates [10], photosynthetic pigments, A_CO2_ [7,10,11], PETC [10], chloroplast ultrastructure [8], oxidative stress [7,10], the transcriptome revealed by cDNA-AFLP [29], and the proteome revealed by two-dimensional electrophoresis (2-DE) [28] in citrus. Additionally, we examined the effects of low pH on growth, uptake of nutrients, photosynthetic pigments, A_CO2_, PETC [40], oxidative stress [41], carbohydrates [42], amino acids, organic acids, the transcriptome revealed by RNA-Seq [43], and the proteome revealed by 2-DE [42] in citrus. However, there is no research on raising pH to reduce Mn toxicity in citrus. For this reason, the research examined the impacts of Mn-pH treatments on seedling growth; nutrient concentrations in roots, stems, and leaves; gas exchange; pigments; and Chl *a* fluorescence (OJIP) transients and related indexes in leaves. The objective was to validate the hypothesis that raising pH reduced Mn toxicity in ‘Sour pummelo’ seedlings by (*a*) reduced uptake of Mn, (*b*) efficient maintenance of other nutrient homeostasis under Mn excess, (*c*) reduced Mn excess-induced impairment of thylakoid structure and PEPC and inhibition of Chl biosynthesis, and (*d*) increased A_CO2_ and subsequent seedling growth under Mn excess.

## 2. Results

### 2.1. Effects of Mn-pH Treatments on Seedling Growth

**Figure 1:**
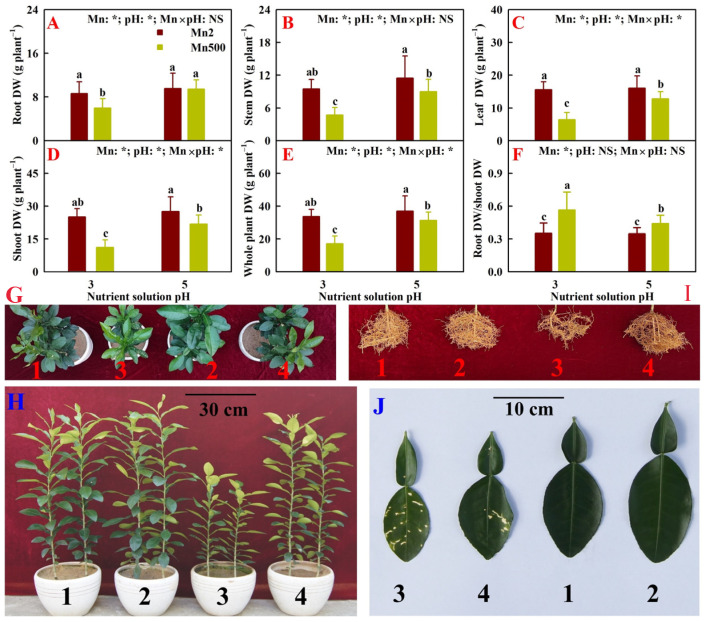
Effects of Mn-pH treatments on the mean (±SD, *n* = 10) root DW (**A**), stem DW (**B**), leaf DW (**C**), shoot DW (**D**), whole plant DW (**E**), and root DW/shoot DW ratio (**F**), as well as growth (**G**–**J**) of ‘Sour pummelo’ seedlings. The bars with different letters indicate significant differences at *p* ≤ 0.05. pH: NS, and Mn × pH: NS indicate that the *F* values for pH and Mn × pH are not significant (*p* > 0.05). Mn: *, pH: *, and Mn × pH: * indicate that the *F* values for Mn, pH, and Mn × pH are significant at *p* ≤ 0.05. 1, Mn2 + P3; 2, Mn2 + P5; 3, Mn500 + P3; and 4, Mn500 + P5.

The current research showed that 500 μM Mn (Mn500) reduced stem, leaf, shoot, and whole plant DW by 50.5% and 21.9%, 58.7% and 20.0%, 55.6% and 20.8%, and 49.3% and 15.7% under pH 3 (P3) and pH 5 (P5), respectively, as well as root DW by 31.0% under P3, but it increased root DW/shoot DW (R/S) by 60.9% and 26.3% under P3 and P5, respectively. Notably, Mn500 did not change root DW under P5. Under Mn500, low pH (P3) reduced root, stem, leaf, shoot, and whole plant DW and increased R/S relative to P5, while pH treatments did not alter them at 2 μM M (Mn2; Figure 1A–F).

Yellowing occurred in young leaves of seedlings treated with Mn500, and necrotic spots occurred in old leaves of seedlings treated with Mn500. Increasing pH reduced Mn500-induced leaf yellowing and necrotic spots (Figure 1G,H,J). Under Mn500, the number of roots reduced and root color became darker. Increasing pH alleviated Mn500-induced reduction in root number and darkening of root color (Figure 1I).

### 2.2. Effects of Mn-pH Treatments on Nutrient Uptake, Distributions, and Balance in Roots, Stems, and Leaves

**Figure 2:**
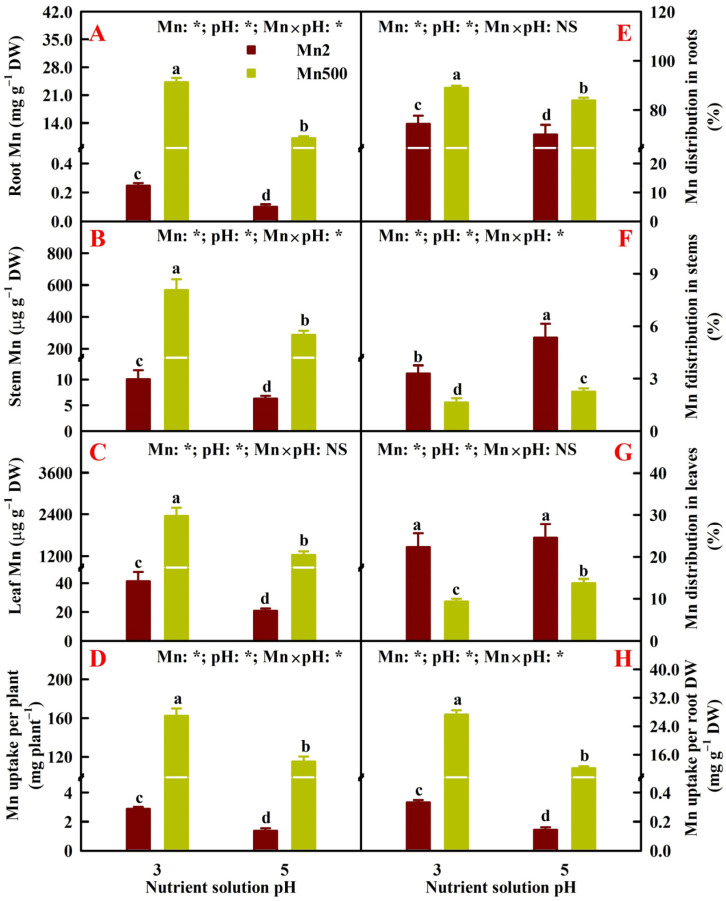
Effects of Mn-pH treatments on the mean (±SD, *n* = 4) Mn concentrations in roots (**A**), stems (**B**), and leaves (**C**); Mn uptake per plant (UPP, (**D**)); Mn distributions in roots (**E**), stems (**F**), and leaves (**G**); and Mn uptake per root DW (UPR, (**H**)) of ‘Sour pummelo’ seedlings. The bars with different letters indicate significant differences at *p* ≤ 0.05. Mn × pH: NS indicates that the *F* values for Mn × pH are not significant (*p* > 0.05). Mn: *, pH: *, and Mn × pH: * indicate that the *F* values for Mn, pH, and Mn × pH are significant at *p* ≤ 0.05.

The research showed that Mn500 elevated Mn concentrations in roots, stems, and leaves; Mn distribution in roots; Mn uptake per plant (UPP); and Mn uptake per root DW (UPR), but it reduced Mn distributions in stems and leaves. Low pH enhanced Mn concentrations in roots, stems, and leaves, Mn distribution in roots, Mn UPP, and Mn UPR, but it lowered Mn distributions in stems and leaves. The only exception was that Mn distributions in leaves at Mn2 were not affected by pH treatments (Figure 2).

**Figure 3:**
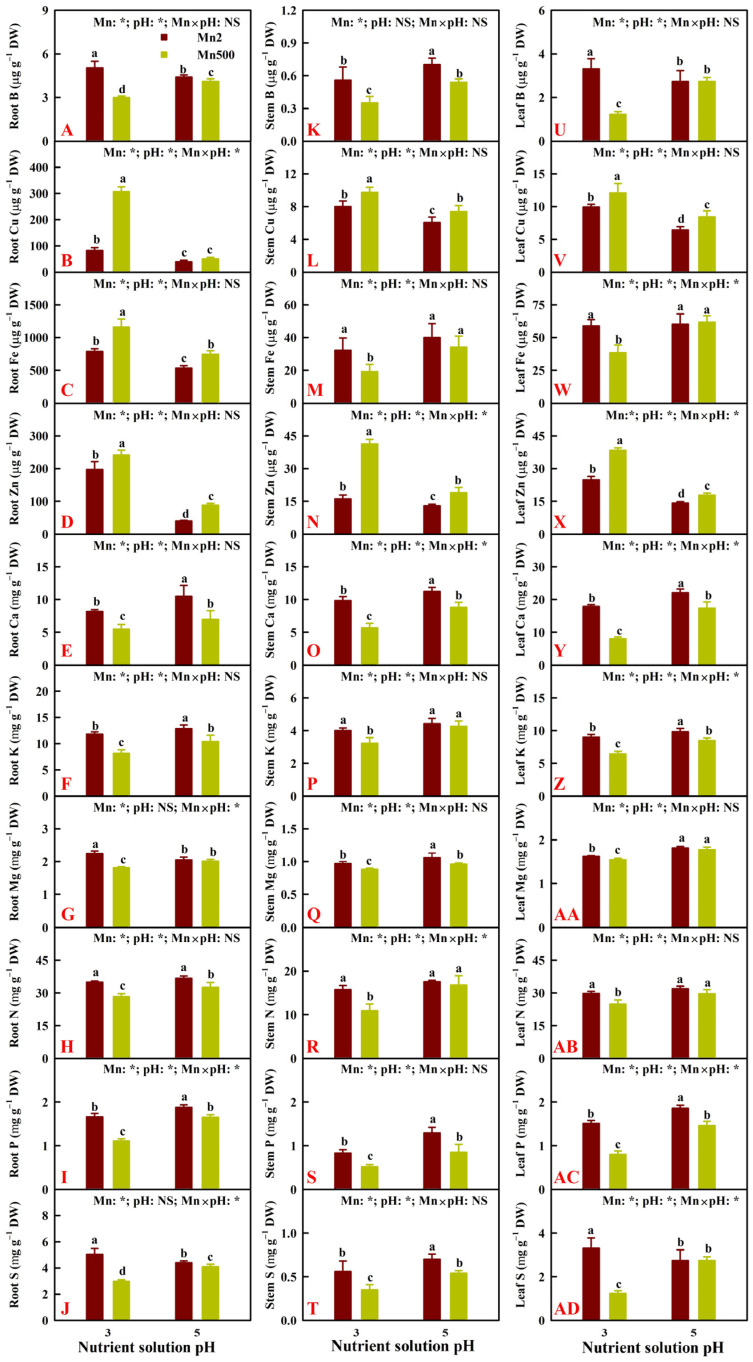
Effects of Mn-pH treatments on the mean (±SD, *n* = 4) concentrations of B, Cu, Fe, Zn, Ca, K, Mg, N, P, and S in roots (**A**–**J**), stems (**K**–**T**), and leaves (**U**–**AD**) of ‘Sour pummelo’ seedlings. The bars with different letters indicate significant differences at *p* ≤ 0.05. pH: NS and Mn × pH: NS indicate that the *F* values for pH and Mn × pH are not significant (*p* > 0.05). Mn: *, pH: *, and Mn × pH: * indicate that the *F* values for Mn, pH, and Mn × pH are significant at *p* ≤ 0.05.

For roots, increasing pH reduced the Mn500-induced decreases in the concentrations of B, Ca, K, Mg, N, P, and S and increases in the concentrations of Cu, Fe, and Zn. The concentrations of B, K, Mg, N, and S were decreased by low pH (P3) at Mn500 but increased or unaffected by P3 at Mn2. Low pH increased the concentrations of Cu, Fe, and Zn and decreased the concentrations of Ca and P at both Mn2 and Mn500 (Figure 3A–J).

For stems, increasing pH alleviated the Mn500-induced decreases in the concentrations of B, Fe, Ca, K, N, P, and S and increase in the concentration of Zn. Notably, the Mn500-induced increase in the concentration of Cu and decrease in the concentration of Mg did not differ between P3 and P5. The concentrations of Fe, K, and N were reduced by P3 at Mn500 but unaffected by P3 at Mn2. Low pH decreased the concentrations of B, Ca, Mg, P, and S and increased the concentrations of Cu and Zn at both Mn2 and Mn500 (Figure 3K–T).

For leaves, increasing pH alleviated Mn500-induced reductions in the concentrations of B, Fe, Ca, K, Mg, N, P, and S, and increment in the concentration of Zn. The exception was that the Mn500-induced increment in the concentration of Cu was not lower at P5 than at P3. The concentrations of B, Fe, N, and S were reduced by P3 at Mn500 but increased or unaffected by P3 at Mn2. Low pH increased the concentrations of Cu and Zn and decreased the concentrations of Ca, K, Mg, and P at both Mn2 and Mn500 (Figure 3U–AD).

**Figure 4:**
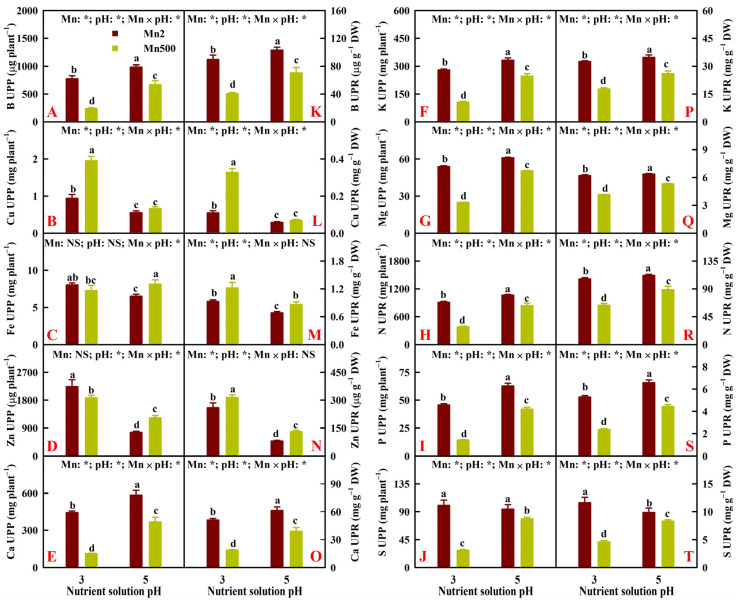
Effects of Mn-pH treatments on the mean (±SD, *n* = 4) nutrient uptake per plant (UPP, (**A**–**J**)) and uptake per root dry weight (UPR, (**K**–**T**)). The bars with different letters indicate significant differences at *p* ≤ 0.05. Mn: NS, pH: NS, and Mn × pH: NS indicate that the *F* values for Mn, pH, and Mn × pH are not significant (*p* > 0.05). Mn: *, pH: *, and Mn × pH: * indicate that the *F* values for Mn, pH, and Mn × pH are significant at *p* ≤ 0.05.

Increasing pH reduced the Mn500-induced decreases in B, Ca, K, Mg, N, P, and S uptake per plant (UPP) and increase in Cu UPP. Notably, Fe and Zn UPP were increased by Mn500 at P5 but increased or unaffected by Mn500 at P3. Low pH decreased B, Ca, K, Mg, N, P, and S UPP but increased Cu and Zn UPP at both Mn2 and Mn500, especially at Mn500. The exceptions were that low pH did not alter S UPP, and low pH increased Zn UPP more at Mn2 than at Mn500 (Figure 4A–J).

Elevating pH alleviated the Mn500-induced reductions in B, Ca, K, Mg, N, P, and S uptake per root DW (UPR) and increases in Cu and Fe UPR. Notably, Mn500 increased Zn UPR more at P5 than at P3. Low pH decreased B, Ca, K, Mg, N, P, and S UPR and increased Cu, Fe, and Zn UPR at both Mn2 and Mn500, especially at Mn500. The exceptions were that low pH increased Zn UPR more at Mn2 than at Mn500, and low pH increased S UPR at Mn2 (Figure 4K–T).

**Figure 5:**
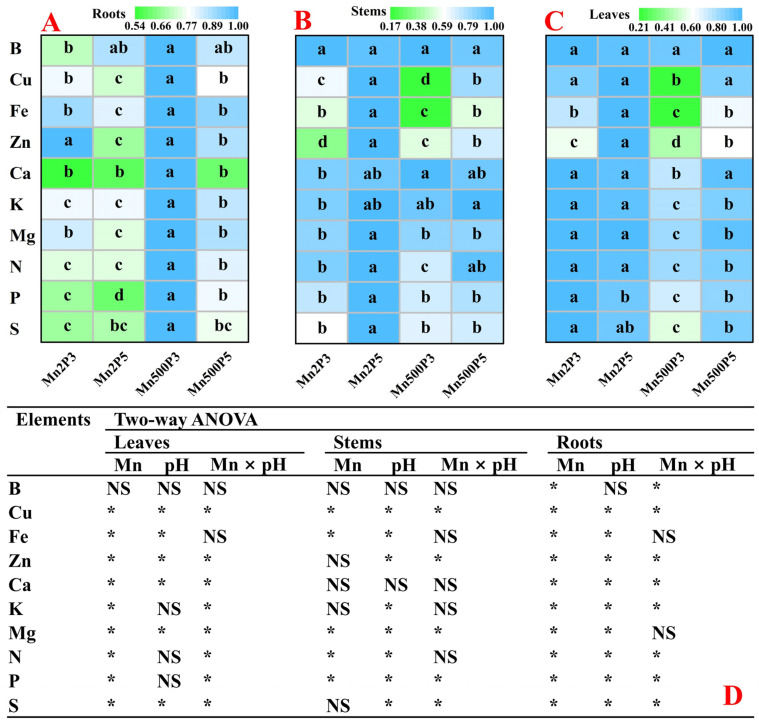
Heatmaps of the other nutrient distributions in roots (**A**), stems (**B**), and leaves (**C**) under Mn-pH treatments, and two-way ANOVA (**D**). Results were the means of 4 replicates. For (**A**–**C**), different letters in the same row for the same tissue indicate a significant difference at *p* ≤ 0.05. For (**D**), NS indicates that the *F* values for Mn, pH, and Mn × pH are not significant (*p* > 0.05); * indicates that the *F* values for Mn, pH, and Mn × pH are significant at *p* ≤ 0.05. Mn2P3, Mn2 + P3; Mn2P5, Mn2 + P5; Mn500P3, Mn500 + P3; Mn500P5, Mn500 + P5.

Mn500 increased or did not affect B, Cu, Fe, Zn, Ca, K, Mg, N, P, and S distributions in roots. Low pH increased or did not alter the distributions of the 10 nutrients in roots (Figure 5A). Mn500 reduced or did not affect the distributions of the 10 nutrients in stems and leaves, with the exception that Mn500 increased Ca distribution in stems at P3. Low pH decreased or did not alter the distributions of the 10 nutrients in stems and leaves, with the exception that low pH increased P distribution in leaves at Mn2 (Figure 5B,C).

**Figure 6:**
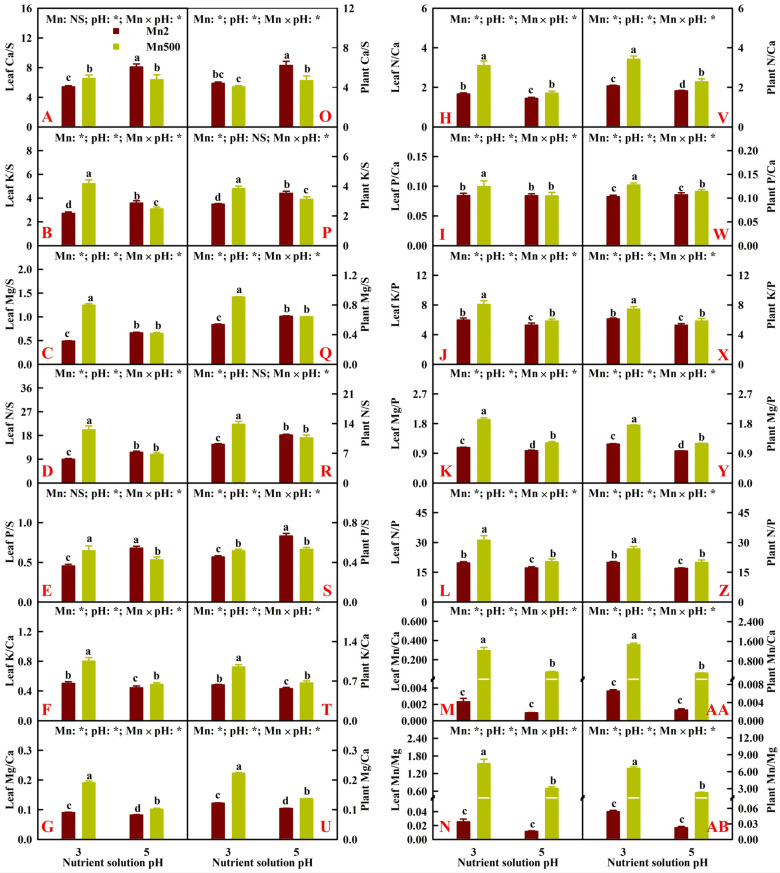
Effects of Mn-pH treatments on the mean (±SD, *n* = 4) ratios of Ca, K, Mg, N, and P concentrations to S concentration, K, Mg, N, and P concentrations to Ca concentration, K, Mg, and N concentrations to P concentration, and Mn concentration to Ca and Mg concentrations in leaves (**A**–**N**), as well as the ratios of Ca, K, Mg, N, and P uptake per plant (UPP) to S UPP, K, Mg, N, and P UPP to Ca UPP, K, Mg, and N UPP to P UPP, and Mn UPP to Ca and Mg UPP (**O**–**AB**). The bars with different letters indicate significant differences at *p* ≤ 0.05. Mn: NS and pH: NS indicate that the *F* values for Mn and pH are not significant (*p* > 0.05). Mn: *, pH: *, and Mn × pH: * indicate that the *F* values for Mn, pH, and Mn × pH are significant at *p* ≤ 0.05.

Figure 6 exhibited the impacts of Mn-pH treatments on the ratios of Ca, K, Mg, N, and P concentrations to S concentration; K, Mg, N, and P concentrations to Ca concentration; K, Mg, and N concentrations to P concentration; and Mn concentration to Ca and Mg concentrations in leaves (hereinafter referred to as leaf Ca/S, K/S, Mg/S, N/S, P/S, K/Ca, Mg/Ca, N/Ca, P/Ca, K/P, Mg/P, N/P, Mn/Ca, and Mn/Mg), as well as the ratios of Ca, K, Mg, N, and P uptake per plant (UPP) to S UPP; K, Mg, N, and P UPP to Ca UPP; K, Mg, and N UPP to P UPP; and Mn UPP to Ca and Mg UPP (hereinafter referred to as plant K/S). The current research showed that leaf Ca/S, K/S, and P/S were increased and decreased by Mn500 at P3 and P5, respectively, and increasing pH reduced the Mn500-induced increases in the other 11 leaf nutrient ratios. Low pH did not affect leaf Ca/S but increased the other 13 leaf nutrient ratios at Mn500, whereas low pH decreased leaf K/Ca, Mg/Ca, N/Ca, K/P, Mg/P, and N/P but slightly increased or did not affect the other 8 leaf nutrient ratios at Mn2 (Figure 6A–N).

The current research showed that plant Ca/S, K/S, and P/S were reduced by Mn500 at P5 but increased or unaffected by Mn500 at P3, and increasing pH reduced the Mn500-induced increases in the other 11 plant nutrient ratios. Low pH decreased plant Ca/S, did not alter plant P/S, and increased the other 12 plant nutrient ratios at Mn50, whereas low pH decreased plant Ca/S, K/S, Mg/S, N/S, and P/S but increased slightly or did not alter the other nine plant nutrient ratios (Figure 6O–AB).

### 2.3. Effects of Mn-pH Treatments on Leaf Pigments and Gas Exchange

**Figure 7:**
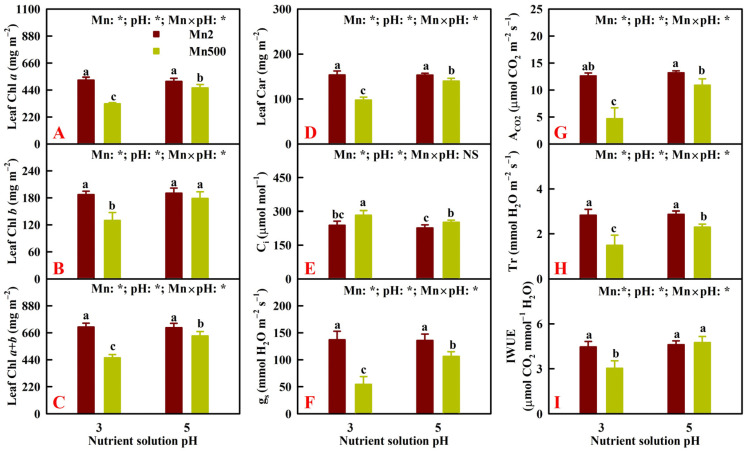
Effects of Mn-pH treatment on the mean (±SD, *n* = 4) Chl *a* (**A**), Chl *b* (**B**), Chl *a* + *b* (**C**), Car (**D**), C_i_ (**E**), g_s_ (**F**), A_CO2_ (**G**), Tr (**H**), and IWUE (I) in leaves. A_CO2_, CO_2_ assimilation; Car, carotenoids; Chl, chlorophyll; C_i_, intercellular CO_2_ concentration; g_s_, stomatal conductance; IWUE, instantaneous water use efficiency; T_r_, transpiration rate. The bars with different letters indicate significant differences at *p* ≤ 0.05. Mn × pH: NS indicates that the *F* value for Mn × pH is not significant (*p* > 0.05). Mn: *, pH: *, and Mn × pH: * indicate that the *F* values for Mn, pH, and Mn × pH are significant at *p* ≤ 0.05.

Mn500 decreased the concentrations of pigments in leaves less at P5 than at P3. Foliar concentrations of pigments were reduced by low pH at Mn500 but unaffected at Mn2 (Figure 7A–D).

Increasing pH mitigated the Mn500-induced decreases in leaf g_s_, A_CO2_, Tr, and IWUE and the increase in leaf C_i_. Low pH decreased leaf g_s_, A_CO2_, Tr, and IWUE and increased C_i_ at Mn500 but did not alter them at Mn2 (Figure 7E–I).

### 2.4. Effects of Mn-pH Treatments on Leaf OJIP Transients and Fluorescence Parameters

**Figure 8:**
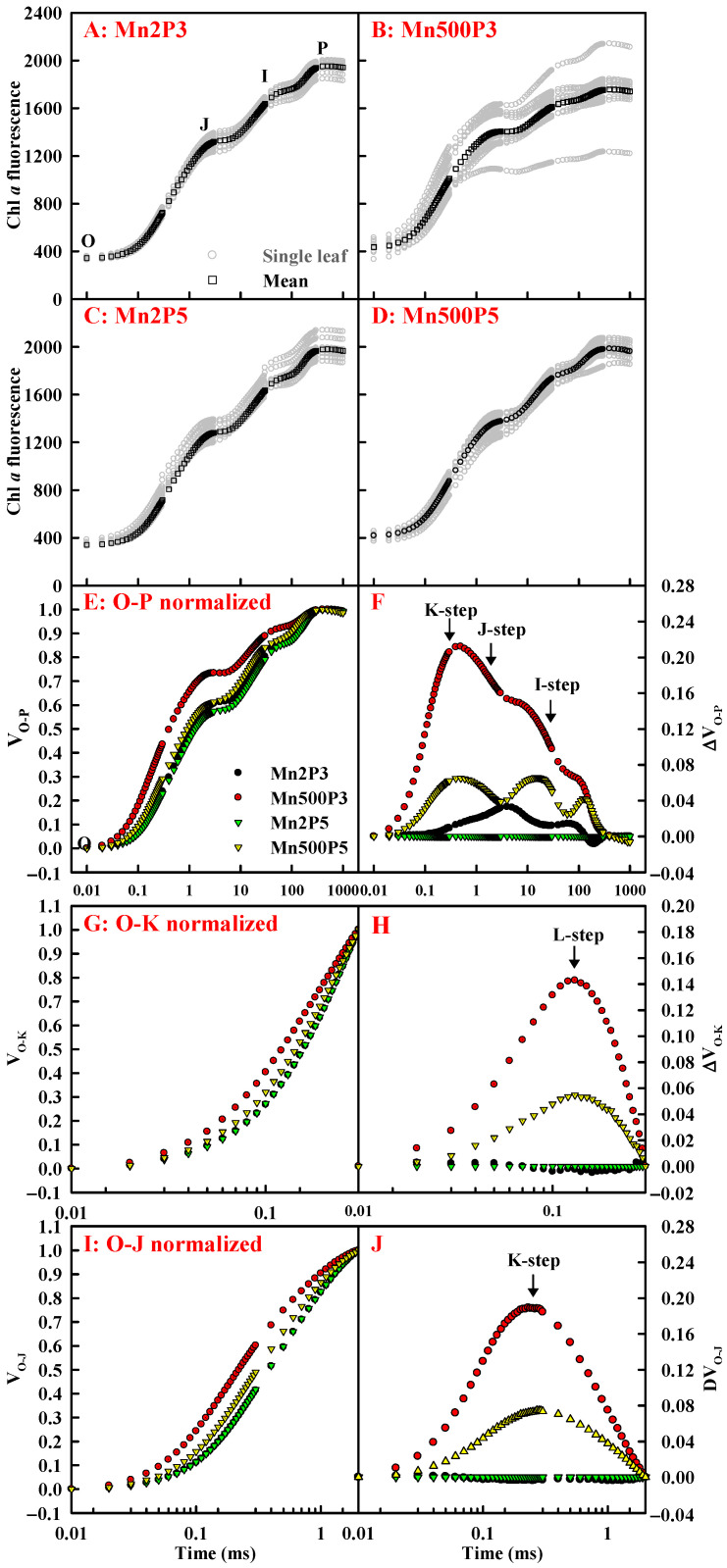
Effects of Mn-pH on OJIP transients of dark-adapted leaves (**A**–**D**), as well as the mean OJIP transients expressed as the kinetics of relative variable fluorescence: between F_o_ and F_m_ (O-P normalized): V_O-P_ = (F_t_ − F_o_)/(F_m_ − F_o_) (**E**) and the differences of the 4 samples to the reference sample treated with Mn2P5 (ΔV_O-P_; (**F**)); between F_o_ and F_300μs_ (O-K normalized): V_O-K_ = (F_t_ − F_o_)/(F_300μ −_ F_o_) (**G**) and the differences of the 4 samples to the reference sample (ΔV_O-K_; (**H**)); and between F_o_ and F_J_ (O-J normalized): V_O-J_ = (F_t_ − F_o_)/(F_J_ − F_o_) (**I**) and the differences of the 4 samples to the reference sample (ΔV_O-J_; (**J**)). F_t_, fluorescence at time *t* after onset of actinic illumination; F_o_, minimum fluorescence; F_m_, maximum fluorescence; F_300μs_, fluorescence intensity at 300 μs; F_J_, fluorescence intensity at the J-step (2 ms).

The research showed that sample heterogeneity was greater at 500 μM Mn + pH 3 (Mn500P3) than at 2 μM Mn + pH 3 (Mn2P3), 2 μM Mn + pH 5 (Mn2P5), and 500 μM Mn + pH 5 (Mn500P5). OJIP transients from leaves of seedlings treated with Mn500P3 displayed elevated O-step and decreased P-step, as well as positive ΔL-step (130 μs), ΔK-step (300 μs), ΔJ-step (2 ms), and ΔI-step (30 ms) relative to OJIP transients from leaves of seedlings treated with Mn2P5. Raising pH reduced Mn500-induced alterations in OJIP transients. Low pH increased sample heterogeneity at Mn500, but the increase was less at Mn2. Low pH had a greater impact on the leaf OJIP transients at Mn500 than at Mn2 (Figure 8).

**Figure 9:**
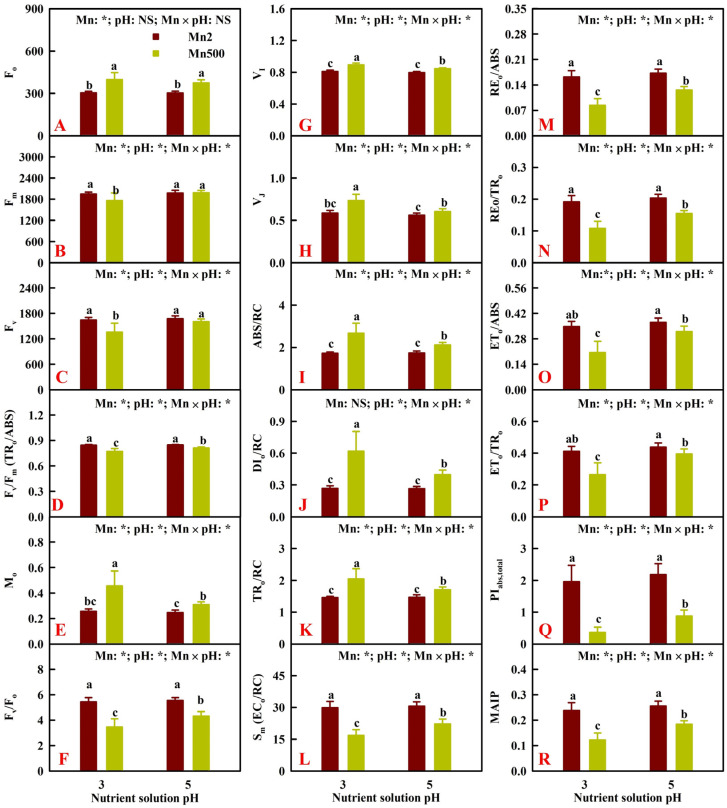
Effects of Mn-pH treatments on the mean (±SD, *n* = 10) F_o_ (**A**), F_m_ (**B**), F_v_ (**C**), F_v_/F_m_ or TR_o_/ABS (**D**), M_o_ (**E**), F_v_/F_o_ (**F**), V_I_ (**G**), V_J_ (**H**), ABS/RC (**I**), DI_o_/RC (**J**), TR_o_/RC (**K**), Sm or EC_o_/RC (**L**), RE_o_/ABS (**M**), RE_o_/TR_o_ (**N**), ET_o_/ABS (**O**), ET_o_/TR_o_ (**P**), PI_abs,total_ (**Q**), and MAIP (**R**) in leaves. F_v_, maximum variable fluorescence; F_v_/F_m_ or TR_o_/ABS, maximum quantum yield of primary photochemistry; M_o_, approximated initial slope (in ms^−1^) of the fluorescence transient *V* = *f*(*t*); F_v_/F_o_, maximum primary yield of photochemistry of photosystem II (PSII); V_I_, relative variable fluorescence at the I-step (30 ms); V_J_, relative variable fluorescence at the J-step (2 ms); ABS/RC, absorption flux per reaction center (RC); DI_o_/RC, dissipated energy flux per reaction center; TR_o_/RC, trapped energy flux per RC; S_m_ or EC_o_/RC, total electron carriers per RC; RE_o_/ABS or φ_Ro_, quantum yield for the reduction in end acceptors of photosystem I per photon absorbed; RE_o_/TR_o_ or ρ_Ro_, efficiency with which a trapped exciton can move an electron into the electron transport chain from Q_A_^−^ to the photosystem I end electron acceptors; ET_o_/ABS or φ_Eo_, quantum yield for electron transport; ET_o_/TR_o_ or ψ_Eo_, probability that a trapped exciton moves an electron into the electron transport chain beyond Q_A_^−^; PI_abs,total_, total performance index; MAIP, maximum amplitude of IP phase. The bars with different letters indicate significant differences at *p* ≤ 0.05. pH: NS and Mn × pH: NS indicate that the *F* values for pH and Mn × pH are not significant (*p* > 0.05). Mn: *, pH: *, and Mn × pH: * indicate that the *F* values for Mn, pH, and Mn × pH are significant at *p* ≤ 0.05.

Increasing pH mitigated the Mn500-induced increase in F_o_, M_o_, V_I_, V_J_, ABS/RC, DI_o_/RC, and TR_o_/RC and the decrease in the other 11 fluorescence indexes. Low pH increased M_o_, V_I_, V_J_, ABS/RC, TR_o_/RC, and DI_o_/RC, did not alter F_o_, and decreased the other 11 fluorescence indexes at Mn500, but it did not change the 18 fluorescence indexes at Mn2 (Figure 9).

### 2.5. Principal Coordinate Analysis, Regression Analysis, and the Different Alterations in Physiological Parameters Between Roots and Leaves Caused by Mn-pH Treatments

**Figure 10:**
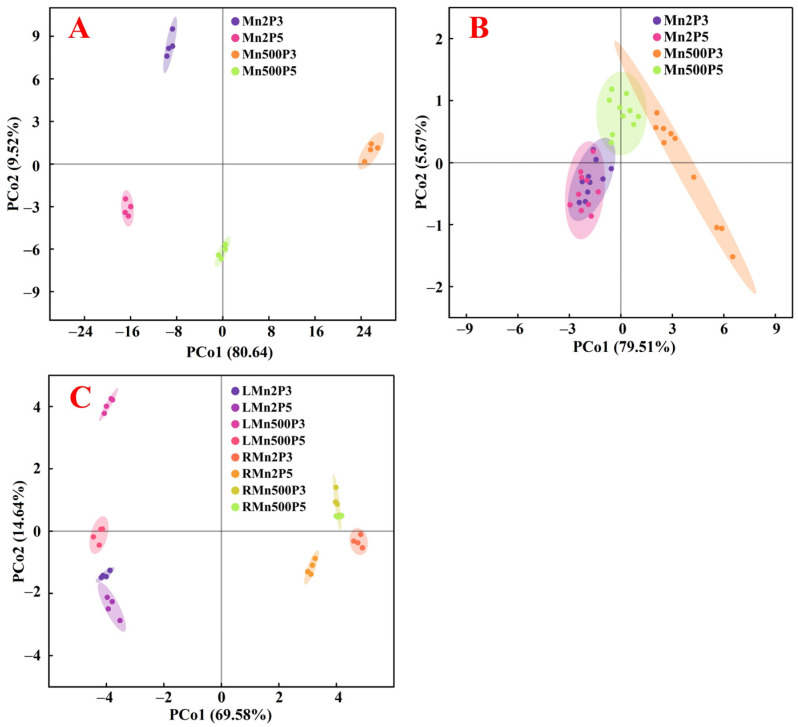
PCoA plots of 125 parameters for nutrients (33 nutrient concentrations, 11 nutrient UPP, 11 nutrient UPR, 33 nutrient distributions, and 28 ratios), pigments (4), gas exchange (5) (**A**), 24 parameters for growth (6) and fluorescence (18) (**B**), and 22 common parameters for roots and leaves (11 nutrient concentrations and 11 nutrient distributions) (**C**) from ‘Sour pummelo’ seedlings exposed to different Mn-pH treatments. LMn2P3, leaves of seedlings treated with Mn2P3; LMn2P5, leaves of seedlings treated with Mn2P5; LMn500P3, leaves of seedlings treated with Mn500P3; LMn500P5, leaves of seedlings treated with Mn500P5; RMn2P3, roots of seedlings treated with Mn2P3; RMn2P5, roots of seedlings treated with Mn2P5; RMn500P3, roots of seedlings treated with Mn500P3; RMn500P5, roots of seedlings treated with Mn500P5.

Principal coordinate analysis was performed using 125 parameters for nutrients, gas exchange, and pigments, as well as 24 parameters for growth and fluorescence (Figure 10A,B). For the 125 parameters, PCo1 and PCo2 clearly separated the effects of Mn and pH treatments, respectively. For the 24 parameters, PCo1 separated the effects of Mn treatments and pH treatments at Mn500. Further analysis indicated that raising pH lowered the impacts of Mn500 on these parameters, as revealed by the shorter distance between Mn500P5 and Mn2P5 than between Mn500P3 and Mn2P3. This was also supported by our findings that Mn500 did not affect 34 (1 index from Figure 1, 10 indexes from Figure 3, 2 indexes from Figure 4, 12 indexes from Figure 5, 5 from Figure 6, 2 from Figure 7, and 2 indexes from Figure 9) out of 149 indexes at P5, but only 9 (1 index from Figure 4, 7 indexes from Figure 5, and 1 index from Figure 6) out of 149 indexes at P3. Additionally, Mn500 intensified the impacts of low pH on these parameters, as revealed by the longer distance between Mn500P5 and Mn500P3 than between Mn2P5 and Mn2P3. This was also supported by our findings that P3 did not alter 63 (6 indexes from Figure 1, 1 index from Figure 2, 7 indexes from Figure 3, 1 index from Figure 4, 15 indexes from Figure 5, 6 indexes from Figure 6, 9 indexes from Figure 7, and 18 indexes from Figure 9) out of 149 indexes relative to P5 at Mn2, but only 11 (8 indexes from Figure 5, 2 indexes from Figure 6, and 1 index from Figure 9) out of 149 indexes relative to P5 at Mn500.

**Figure 11:**
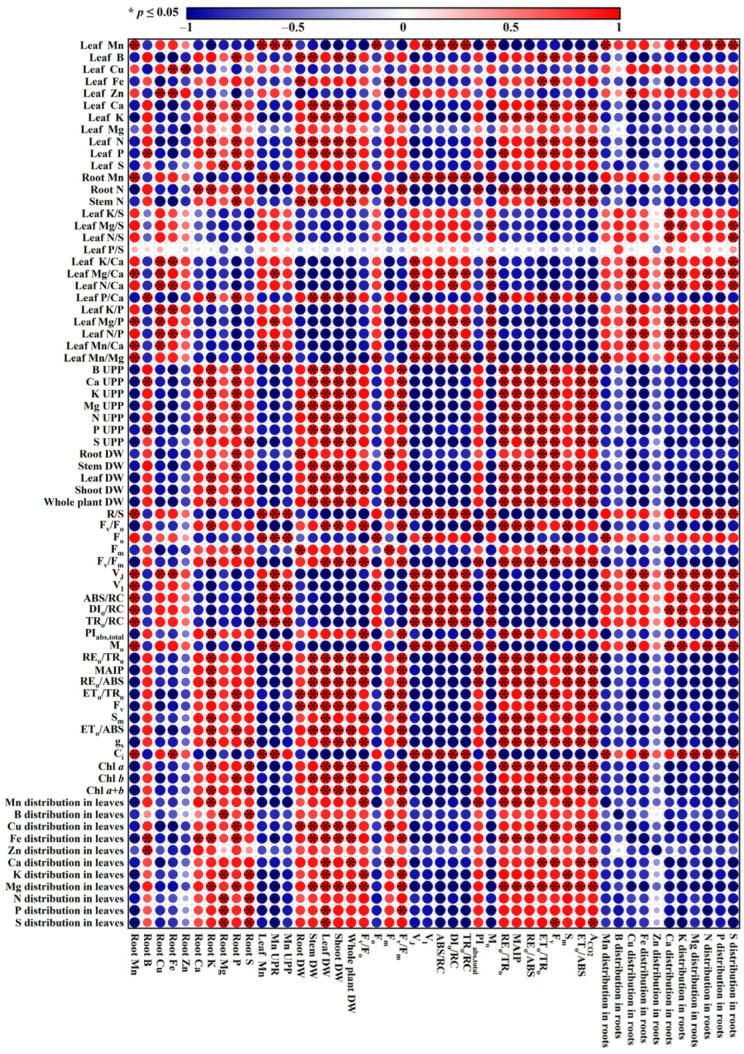
Pearson’s correlation coefficient matrix for the mean values of some parameters.

Principal coordinate analysis was performed using the 22 common parameters (nutrient concentrations and distributions) present in both leaves and roots (Figure 10C). The results indicated that the parameters for roots and leaves were clustered on the right and left sides, respectively, meaning that the responses of root and leaf parameters to Mn-pH treatments differed. This was also supported by the findings that among the 22 common parameters present in both leaves and roots, 9 parameters (Mn, B, Cu, Fe, Zn, Ca, K, Mg, and S distributions in leaves) in leaves were significantly negatively correlated with the corresponding parameters in roots (Figure 11 and Table S1). Mn500 increased N and P distributions in roots, but it lessened N and P distributions in leaves, with the exception that Mn500 did not change P distribution in leaves at pH 5 (Figure 5). Additionally, Mn500 increased Fe concentration in roots, but it decreased or did not alter the Fe concentration in leaves (Figure 3C,W).

Principal coordinate analysis showed that the four treatments for leaves (LMn2P3, LMn2P5, LMn500P3, and LMn500P5) were more separated than the four treatments for roots (RMn2P3, RMn2P5, RMn500P3, and RMn500P5) (Figure 10C), implying that Mn-pH treatments had a greater impact on the 22 parameters in leaves than in roots. This was supported by the view that Mn toxicity is primarily expressed in plant shoots [2].

## 3. Discussion

### 3.1. Increased pH-Mediated Mitigation of Mn500 in ‘Sour Pummelo’ Involved Lessened Uptake of Mn Rather than Elevated Mn Distribution in Roots

We found that Mn500 increased the distribution of Mn in roots (Figure 2H), as obtained on *C. sinensis* (L.) Osbeck, *C. grandis* (L.) Osbeck [28], and trifoliate orange [11]. Reducing root-to-shoot Mn transport can enhance plant tolerance to Mn toxicity [44]. However, raised pH-mediated mitigation of Mn500 in ‘Sour pummelo’ seedlings could not be explained by the elevated Mn distribution in roots, because the distribution of Mn in roots of seedlings treated with Mn500 was lower at P5 than at P3 (Figure 2H). This agreed with the findings that raised pH-mediated mitigation of Mn excess in white clover was accompanied by decreased Mn level in roots and unaltered Mn level in shoots [35] and that H_2_S-mediated amelioration of Mn excess in *M. hupehensis* (Pamp.) Rehder was accompanied by decreased Mn uptake and increased percentage of Mn in leaves [25]. However, Noor et al. [6] observed that P-mediated amelioration of Mn excess in peach involved lessened Mn uptake and increased fixation of Mn in roots, because P reduced Mn excess-induced increase in Mn level more in leaves than in roots. In addition to lowering root-to-shoot Mn transport, reduced uptake of Mn also contributes to the resilience of plants to Mn excess [5,27,30]. The current research showed that both the Mn uptake and the Mn concentration in roots, stems, and leaves were lower at P5 than P3 (Figure 2A–E). This agreed with the reports that soil liming alleviated Mn excess in *Juglans regia* L., *Robinia pseudoacacia* L., *Populus* sp., and *Eucalyptus* sp. by increasing soil pH and reducing the diethylenetriaminepentaacetic acid-extractable Mn concentration in soil and the Mn level in leaves from trees with Mn toxicity symptoms before liming [45] and that the alleviation of Mn toxicity in peanut mediated by NaHCO_3_ and lime involved increased pH and reduced leaf Mn concentration [22]. Previous studies showed that P, malate, taurine, and coumarin can alleviate Mn toxicity in peach [6], *S. guianensis* (Aubl.) Sw. [30], *T. alexandrinum* L. [20], and *Sesamum indicum* L. [46], respectively, by reducing Mn uptake. Regression analysis showed that Mn uptake per root DW (UPR) was significantly negatively related with leaf, shoot, and whole plant DW (Figure 11 and Table S1). These findings indicated that increasing pH reduced the bioavailability and uptake of Mn, thus alleviating Mn toxicity of ‘Sour pummelo’ seedlings.

### 3.2. Increased pH-Mediated Mitigation of Mn500 in ‘Sour Pummelo’ Involved Efficient Maintenance of Nutrient Homeostasis

The current research indicated that increasing pH alleviated Mn500-induced decreases in B, Ca, K, Mg, N, P, N, and S uptake per plant (UPP) and per root DW (UPR) (Figure 4). Also, increasing pH mitigated Mn500-induced decreases in B, Ca, K, Mg, N, P, and S levels in roots, stems, and leaves (Figure 3). This might be related to the findings that increasing pH counteracted Mn500-induced impairment of roots, as revealed by the unchanged root DW (Figure 1A). Hafeez et al. [20] observed that excessive Mn led to a considerable decreases in Ca, K, and P levels and an increment in Mn level in *T. alexandrinum* L. plants, suggesting that poor nutrient acquisition was responsible for the excessive Mn-induced decrement of plant growth. Shad et al. [46] reported that coumarin-mediated alleviation of Mn excess in *S. indicum* L. involved elevated acquisition of Fe, Zn, Ca, K, Mg, and N. Plant Mn excess can be mitigated by application of P [6,21], Fe [26], and lime [23]. Regression analysis indicated that stem, leaf, shoot, and whole plant DW were significantly positively related with B, Ca, K, Mg, N, P, and S UPP, with the exception of the relationship between S UPP and stem DW (*r* = 0.9323), and that root, stem, leaf, shoot, and whole plant DW were significantly positively related with root, stem, and leaf N concentration, with the exception of relationships between root N concentration and root DW (*r* = 0.8572), stem N concentration and leaf DW (*r* = 0.9253), and stem N concentration and shoot DW (*r* = 0.9473) (Figure 11 and Table S1). Notably, Mn500 did not lower Fe UPP and UPR at both P3 and P5 (Figure 4), but it lessened and did not affect the Fe level in stems and leaves and increased the Fe level and distribution in roots at P3 and P5 (Figure 3 and Figure 5). These results indicated that Mn500 prevented root-to-shoot Fe translocation in ‘Sour pummelo’ seedlings. This was supported by the report that Mn excess elevated and lowered the Fe level in soybean roots and leaves, respectively [16].

It was found that Mn500 decreased Ca and Mg uptake per plant (UPP) and per root DW (UPR) (Figure 4) and their levels in roots, stems, and leaves (Figure 3), but it increased Cu UPP and UPR and its level in roots, stems, and leaves (Figure 2), as well as leaf and plant Mn/Mg and Mn/Ca (Figure 6), especially at P3. This agreed with the reports that Mg and Ca compete with Mn for plant uptake [47], and Cu has a synergistic effect on Mn uptake [48]. Notably, among the macronutrients, Mn500 reduced leaf S concentration the most, followed by leaf Ca and P concentration at P3 (Figure 3). However, Mn500 led to a similar reduction in S and Ca uptake per plant (UPP), followed by P UPP at P3 (Figure 4). Therefore, Mn500 greatly disturbed macronutrient balance, especially at P3 (Figure 6). This agreed with the report that Mn excess caused nutrient imbalance in plants via interacting with other nutrients and their related metabolic processes [20]. Regression analysis showed that root, stem, leaf, shoot, and whole plant DW were significantly negatively related with plant K/Ca, Mg/Ca, N/Ca, P/Ca, K/P, Mg/P, N/P, Mn/Ca, and Mn/Mg, with the exception of relationships between root DW and plant N/Ca (*r* = −0.9441), root DW and plant P/Ca (*r* = −0.8107), root DW and plant Mn/Ca (*r* = −0.9271), and root DW and plant Mn/Mg (*r* = −0.8754) (Figure 11 and Table S1). Application of NaHCO_3_ reduced Mn toxicity in peanut plants by lowering Mn level and increasing Mg/Mn in leaves [22]. Hue et al. [32] reported that Ca competed with Mn for uptake, high Ca conferred plant Mn tolerance, and the correlation between soybean DW and leaf Ca/Mn was better than the correlation with leaf Mn concentration.

The current research indicated that R/S was significantly positively related with K, N, P, and S distributions in roots and showed a raised trend with the increased distributions of Ca (*r* = 0.9409) (Figure 11 and Table S1), and that except for unaltered Zn distribution in roots, Mn500 increased the distributions of the other nine nutrients in roots, especially at P3 (Figure 5), implying that more nutrients were allocated to roots of seedlings treated with Mn500 to support their growth, thereby upregulating R/S. Zhou et al. [29] observed that Mn excess led to a greater increase in R/S in Mn-tolerant *C. sinensis* (L.) Osbeck than in Mn-sensitive *C. grandis* (L.) Osbeck. An increment in R/S might be an adaptive strategy to Mn excess, because relatively fewer shoots were fed by relatively more roots with nutrients.

Taken together, increasing pH lessened Mn500-induced impairment of roots and interference of nutrient uptake, thereby improving nutrient acquisition and maintaining nutrient balance, thus endowing ‘Sour pummelo’ with Mn tolerance.

### 3.3. Increasing pH Mitigated Mn500-Induced Decline in Leaf A_CO2_ and Subsequent Decrease in Seedling Growth

The research showed that A_CO2_ was significantly positively related to g_s_ (Figure 11 and Table S1), and increasing pH mitigated Mn500-induced decline in A_CO2_ (Figure 7G), but this did not necessarily imply that elevated pH-mediated amelioration of A_CO2_ decline in leaves of seedlings treated with Mn500 was mainly caused by stomatal limitation, because A_CO2_ was significantly negatively related to C_i_. Although A_CO2_ was significantly positively related to Chl *a*, Chl *b*, or Chl *a* + *b* level (Figure 11 and Table S1), elevated pH-mediated amelioration of A_CO2_ decrease in leaves of seedlings treated with Mn500 was not caused by the increases in their levels, because the decreases in their levels in response to Mn500 were smaller than that of A_CO2_ (Figure 7). This was supported by the observation that A_CO2_ was significantly negatively related to DI_o_/RC (Figure 11 and Table S1) and the leaves of seedlings treated with Mn500 had a higher DI_o_/RC at P3 than at P5 (Figure 9).

The current study showed that increasing pH resisted the fall in F_v_/F_o_ (an indicator of structural damage to thylakoids) in LMn500 (Figure 9). Regression analysis showed that F_v_/F_o_ was significantly negatively related to leaf Mn/Mg or Mn concentration and had a reduced trend with the decrease in leaf Fe concentration and that A_CO2_ had a lessened trend with the decrease in F_v_/F_o_ (Figure 11 and Table S1). Mn can replace Mg in Chl molecules or bind to ferredoxin in the thylakoid matrix, ultimately damaging chloroplast ultrastructure. Fe deficiency may hinder the biosynthesis of Chl and the correct assembly of photosystem I (PSI) [49]. A study indicated that P mitigated Mn excess-induced disruption of chloroplast ultrastructure and A_CO2_ in peach leaves [6]. These results demonstrated that increasing pH ameliorated Mn500-induced impairment of thylakoid structure in leaves caused by Fe deficiency and competition of Mn with Mg, thus resisting the fall in A_CO2_.

The research showed that increasing pH counteracted Mn500-induced decreases in ET_o_/ABS and F_v_/F_m_, increase in DI_o_/RC (Figure 9), and alterations in OJIP transients (Figure 8), suggesting that Mn500 caused severe photoinhibition damage to PSII complexes at P3, but the damage was milder at P5 [50]. This agreed with the report that excessive Mn caused photoinhibition of PSII, thereby lowering A_CO2_ in *C. grandis* (L.) Osbeck leaves [10]. Mn500-induced decrement in F_v_/F_m_ at P3 was caused by both an increase in F_o_ and a decrease in F_m_ (Figure 9). The increment in F_o_ might be associated with the accumulation of reduced OA^-^, as shown by the increment in M_o_ (Figure 9) and/or the impairment of the oxygen-evolving complex (OEC), as revealed by the positive K-step at ~300 μs (an indicator of impairment to OEC; Figure 8J). Increasing pH mitigated the Mn500-induced increments in the positive K-step (Figure 8), F_o_, and M_o_ and the decline in F_m_ (Figure 9). The current research indicated that increasing pH counteracted Mn500-induced striking positive L-, J-, and I-steps (Figure 8) and decreased MAIP in OJIP transients (Figure 9). The positive L-step at ~130 μs in the OJIP transients from leaves of seedlings treated with Mn500 indicated that the PSII unit from leaves of seedlings treated with Mn500 had lower energy transfer and absorption efficiency [51] and became less stable and more fragile [10], especially at P3. The positive J-step (V_J_) and I-step (V_I_) (Figure 8), as well as decreased MAIP (Figure 9), implied that the Mn500-induced impairment of the PSII acceptor side was greater than that of the PSII donor side [10], especially at P3. This agreed with the Mn500-induced decline in F_v_ and increase in F_o_, the photoinhibitory damage characteristics on an PSII acceptor side [52].

The current research showed that increasing pH resisted the decreases in PI_abs,total_, MAIP, RE_o_/TR_o_, and RE_o_/ABS caused by Mn500 (Figure 9), suggesting that increasing pH mitigated Mn500-induced impairment to the reduction in PSI end-acceptors. Regression analysis showed that A_CO2_ was significantly positively (negatively) related to F_v_/F_m_, RE_o_/TR_o_, MAIP, ET_o_/TR_o_, RE_o_/ABS, F_v_, or ET_o_/ABS (V_J_, V_I_, ABS/RC, DI_o_/RC, TR_o_/RC, or M_o_) and displayed an increased (a lessened) trend with the rise of F_m_, PI_abs,total_, F_v_/F_o_, or S_m_ (F_o_) (Figure 11 and Table S1). These results suggested that increasing pH reduced Mn500-induced damage of whole PETC, thus resisting the decline in A_CO2_ under Mn500.

Also, both nutrient deficiencies and imbalances can disturb PETC and lessen A_CO2_ [51,53,54,55]. Regression analysis showed that A_CO2_ was significantly positively related to B, Ca, K, N, or P concentration in leaves, as well as B, Ca, K, N, P, or S uptake per plant (UPP), and negatively related to leaf Mg/S, K/Ca, Mg/Ca, N/Ca, P/Ca, K/P, Mg/P, N/P, Mn/Ca, or Mn/Mg. Additionally, F_m_, F_v_/F_m_, PI_abs,total_, RE_o_/TR_o_, MAIP, F_v_/F_o_, RE_o_/ABS, ET_o_/TR_o_, F_v_, S_m_, or ET_o_/ABS significantly elevated or displayed an increased trend with the increment in leaf B, Fe, Ca, K, Mg, N, P, or S concentrations, as well as B, Ca, Mg, K, N, P, or S UPP, but significantly decreased or showed a lessened trend with the increment in leaf K/S, Mg/S, N/S P/S, K/Ca, Mg/Ca, N/Ca, P/Ca, K/P, Mg/P, N/P, Mn/Ca, or Mn/Mg; F_o_, V_J_, V_I_, ABS/RC, DI_o_/RC, TR_o_/RC, or M_o_ significantly decreased or displayed a decreased trend with the increment in leaf B, Fe, Ca, K, Mg, N, P, or S concentrations, as well as B, Ca, Mg, K, N, P, or S UPP, but significantly increased or showed a raised trend with the increment in leaf K/S, Mg/S, N/S P/S, K/Ca, Mg/Ca, N/Ca, P/Ca, K/P, Mg/P, N/P, Mn/Ca, or Mn/Mg (Figure 11 and Table S1). These results revealed that increasing pH enhanced the ability of leaves of seedlings treated with Mn500 to acquire nutrients and maintain nutrient balance, thereby resisting Mn500-induced damage to PETC and decline in A_CO2_.

The research indicated that A_CO2_ was significantly positively related to stem, leaf DW, shoot, or whole plant DW, and had an increased trend with the rise of root DW (Figure 11 and Table S1). Santos et al. [56] indicated that Mn excess lowered photosynthetic rate in soybean leaves and subsequently dry mass production. These results revealed that raising pH counteracted Mn500-induced decline in A_CO2_ and subsequent decrease in seedling growth.

## 4. Materials and Methods

### 4.1. Plant Materials

Seedling culture and Mn-pH treatments were performed as given by Zhou et al. [29] with some modifications. In mid-March 2023, uniform seeds of ‘Sour pummelo’ (*Citrus grandis* (L.) Osbeck) were chosen to sow in plastic seedling trays filled with sand. In mid-May, uniformly sized 6-week-old seedlings were transported to 6 L pots (two seedlings per pot) containing sand. Then, they were grown under natural conditions in an unheated greenhouse at Fujian Agriculture and Forestry University, Fuzhou (26°5′ N, 119°14′ E), with an annual average relative humidity, temperature, and sunshine hours of ~76%, 20 °C, and 1600 h, respectively [37]. After 7 weeks of seedling transplantation, they were supplied six times per week with freshly prepared nutrient solution until dripping (~500 mL per pot) for 25 weeks. The nutrient solution contained 1.25 mM KNO_3_, 0.5 mM MgSO_4_, 1 mM Ca(NO_3_)_2_, 0.25 mM (NH_4_)H_2_PO_4_, 0.25; 10 μM H_3_BO_3_, 20 μM Fe-EDTA, 2 μM ZnCl_2_, 0.065 μM (NH_4_)_6_Mo_7_O_24_, and 0.5 μM CuSO_4_ at a Mn concentration of 2 (Mn2) or 500 (Mn500 or Mn excess) μM from MnCl_2_ (AR, from Sinopharm Chemical Reagent Shanghai Co., Ltd., Shanghai, China) and a pH of 5 (P5) or 3 (P3; adjusted with 1M HCl before supply). There were four treatments, each treatment with 16 pots (32 seedlings) in a completely randomized design. At the end of the experiment, the most recent fully expanded (~7-week-old) leaves were used for all measurements. After leaf OJIP transient and gas exchange were measured in late December, leaf disks from the same seedlings (one seedling per pot) were collected and immediately immersed in liquid N_2_ and then stored in −80 °C freezer until extraction of pigments. These seedlings without collected leaf discs (one seedling per pot) were used to measure nutrients and biomass.

### 4.2. Measurements of Leaf Gas Exchange and OJIP Transient

Intercellular CO_2_ concentration, A_CO2_, Tr, and g_s_ were measured with a CIRAS-2 portable photosynthesis system (PP Systems, Hitchin, Herts, UK) between 9:00 and 11:00 a.m. [57]. IWUE was calculated as A_CO2_/Tr [54].

Leaf OJIP transients were measured at room temperature (~25 °C) on plants adapted to darkness for 3 h with a Handy PEA (Hansatech Instruments Limited, King’s Lynn, Norfolk, UK). All fluorescence indexes were calculated as described by Jiang et al. [58] and Kalaji et al. [51]. Table S2 listed the parameters extracted and calculated from the OJIP transient.

### 4.3. Measurements of Biomass, Pigments, and Nutrients

Stem, leaf, and root DW were weighted after being dried at ~70 °C to a constant weight [57].

Leaf Car, Chl *a*, and Chl *b* were extracted with 80% (*v*/*v*) acetone and then determined, as described by Arnon [59] and Lichtenthaler [60]. The calculation of Chl *a*, Chl *b*, and Car was as follow:

Chl *a* (mg L^−1^) = 12.7 × A_663_ − 2.69 × A_645_

Chl *b* (mg L^−1^) = 22.9 × A_645_ − 4.68 × A_663_

            Car (mg L^−1^) = (1000 × A_470_ − 1.82 × Chl *a* − 85.02 × Chl *b*)/198

The recent fully expanded mature leaves, the fibrous roots, and the middle sections of stems were collected for the determination of nutrients [57]. For the assay of Mn, Cu, Fe, Zn, Ca, K, Mg, P, and S, samples were digested in a mixture of HNO_3_:HClO_4_ (5:1; *v*/*v*) [55]. Mn, Cu, Fe, Zn, Ca, and Mg were measured using a PinAAcle 900F Atomic Absorption Spectrometer (Perkinelmer Singapore Pte Ltd., Singapore) [55]. K was assayed with an FP640 Flame Photometry (Shanghai Precision Scientific Instrument Co., Ltd., Shanghai, China) [40]. P was determined colorimetrically as blue molybdate-phosphate complexes [55]. S was measured using the simple turbidimetric method based on the formation of BaSO_4_ precipitate in colloid form [55]. After ashing the sample at 500 °C for 5 h and dissolving it in 0.1 M HCl, B in the solution was determined by the curcumin method [61]. N was measured by indophenol blue spectrophotometry (Forestry Industry Standards of the People’s Republic of China; LY/T 1269-1999 [55]).

The uptake and distributions of nutrients were calculated as given by Long et al. [40]. Nutrient uptake per plant was the sum of the nutrient content (element concentration × tissue DW) in the roots, stems, and leaves. Nutrient uptake per root DW was calculated as follows: (the sum of the nutrient content in the roots, stems, and leaves)/root DW. Nutrient distributions in roots, stems, or leaves (%) were calculated as follows: (nutrient content in roots, stems, or leaves/the sum of nutrient content in roots, stems, and leaves) × 100.

### 4.4. Statistical Analysis

Means were tested by two-way ANOVA followed by the least significant difference (LSD) at *p* ≤ 0.05 using DPS 7.05 (Hangzhou Ruifeng Information Technology Co., Ltd., Hangzhou, China). The calculation of Pearson correlation coefficients was performed with the SPSS statistical software (version 17.0, IBM, Armonk, NY, USA). Principal coordinate analysis (PCoA) was performed with the ChiPlot (https://www.chiplot.online/ (accessed on 1 March 2025)).

## 5. Conclusions

Increasing pH conferred ‘Sour pummelo’ tolerance against Mn toxicity by (*a*) reduced Mn uptake; (*b*) increased other nutrient (B, Ca, K, Mg, N, P, and S) acquisition and efficient maintenance of nutrient balance under Mn500; (*c*) reduced Mn excess-induced impairment of thylakoid structure and PEPC and inhibition of Chl biosynthesis; and (*d*) enhanced A_CO2_ and subsequent seedling growth under Mn500. The research results can provide a foundation for subsequent studies on improving pH to alleviate HM (Mn) toxicity, as well as a theoretical basis for developing acidic soil amendments and mitigating HM (Mn) toxicity, although the molecular mechanisms still need to be revealed. In order to reasonably utilize lime or other methods to increase soil pH and protect citrus from HM (Mn) toxicity, large-scale field experiments are also needed.

## Data Availability

Data are archived in L.-S. Chen’s laboratory and available upon request.

## Supplementary Materials

The following supporting information can be downloaded at: https://www.mdpi.com/article/10.3390/plants14152390/s1, Table S1: Pearson correlation coefficient matrix for the mean values of 149 physiological parameters in four Mn-pH treatments; Table S2: Summary of parameters, formulae, and their description using data extracted from chlorophyll (Chl) *a* (OJIP) transient.
