# Supplementary material for: Raising pH Reduces Manganese Toxicity in Citrus grandis (L.) Osbeck by Efficient Maintenance of Nutrient Homeostasis to Enhance Photosynthesis and Growth
Source: Plants (Basel). 2025 Aug 2;14(15):2390. doi: 10.3390/plants14152390 (PMC12349334; doi:10.3390/plants14152390)
Supplement: Supplementary file 1 [file plants-14-02390-s001.zip › plants-3772015.pdf]

Table S2: Summary of parameters, formulae and their description using data extracted from chlorophyll (Chl) *a* (OJIP) transient.

F_o	Minimum fluorescence, when all PSII RCs are open
F_m	Maximum fluorescence, when all PSII RCs are closed
$F_v = F_m - F_o$	Maximum variable fluorescence
F_J	Fluorescence intensity at the J-step (2 ms)
F_I	Fluorescence intensity at the I-step (30 ms)
Area	Total complementary area between fluorescence induction curve and $F = F_m$
$\varphi_{Po} = TR_o/ABS = 1 - F_o/F_m = F_v/F_m$	Maximum quantum yield of primary photochemistry at $t = 0$
$M_o = 4\ (F_{300\mu s} - F_o)/(F_m - F_o)$	Approximated initial slope (in ms^{-1}) of the fluorescence transient $V = f(t)$
$F_v/F_o = (F_m - F_o)/F_o$	Maximum primary yield of photochemistry of photosystem II (PSII)
$V_I = (F_{30ms} - F_o)/(F_m - F_o)$	Relative variable fluorescence at the I-step (30 ms)
$V_J = (F_{2ms} - F_o)/(F_m - F_o)$	Relative variable fluorescence at the J-step (2 ms)
$ABS/RC = M_o/V_J/\varphi_{Po}$	Absorption flux per RC
$DI_o/RC = ABS/RC - TR_o/RC$	Dissipated energy flux per RC at $t = 0$
$TR_o/RC = M_o/V_J$	Trapped energy flux per RC at $t = 0$
$S_m = EC_o/RC = Area/(F_m - F_o)$	Normalized total complementary area above the OJIP (reflecting multiple-turnover Q_A reduction events) or total electron carriers per RC
$\varphi_{Ro} = RE_o/ABS = \varphi_{Po} * \psi_{Eo} * \delta_{Ro}$	Quantum yield for the reduction of end acceptors of PSI per photon absorbed
$\rho_{Ro} = RE_o/TR_o = \psi_o * \delta_{Ro}$	Efficiency with which a trapped exciton can move an electron into the electron transport chain from Q_A^- to the PSI end electron acceptors
$\varphi_{Eo} = ET_o/ABS = F_v/F_m * (1 - V_J)$	Quantum yield for electron transport at $t = 0$
$\psi_{Eo} = ET_o/TR_o = 1 - V_J$	Probability (at time 0) that a trapped exciton moves an electron into the electron transport chain beyond Q_A^-
$PI_{abs,total} = (RC/ABS)*(\varphi_{Po}/(1-\varphi_{Po})) *(\psi_{Eo}/(1-\psi_{Eo})) *(\delta_{Ro}/(1-\delta_{Ro}))$	Total performance index, measuring the performance up to the PSI end electron acceptors
$MAIP = (F_m - F_o)/(F_I - F_o) - 1$	Maximum amplitude of IP phase
